# “I Haven’t Had Anyone Talk to Me About Phone Use, At All”: Primiparous Mothers’ Conversations About Smartphone Use While Caregiving

**DOI:** 10.3390/ijerph22101580

**Published:** 2025-10-17

**Authors:** Miriam McCaleb, Rachael Dixon, Patricia Champion, Philip J. Schluter

**Affiliations:** 1 School of Health Sciences, University of Canterbury (Te Whare Wānanga o Waitaha), Christchurch 8041, New Zealandphilip.schluter@canterbury.ac.nz (P.J.S.); 2 Department of Psychological Medicine, Otago University, Dunedin 8011, New Zealand; 3 Primary Care Clinical Unit, School of Clinical Medicine, The University of Queensland, Brisbane 4072, Australia

**Keywords:** smartphone, technoference, infants, transition to parenthood, perinatal, Aotearoa|New Zealand

## Abstract

Our smartphone habits have implications for our mental health, including new mothers’ experience of loneliness. For a baby, whose developmental trajectory will be directly impacted by their attachment relationship, a parent’s unconscious smartphone use is likely to be of lifelong consequence given the impact of such use on attachment. Therefore, new parents would benefit from support in assessing their smartphone habits and the impact on their relationships with their infants. This raises the question—are they receiving any such advice or guidance from perinatal health professionals? This study describes findings from semi-structured interviews and subsequent reflexive thematic analysis with primiparous women in Aotearoa|New Zealand. We found that the women in this sample had had very few discussions about their smartphone use initiated by the perinatal workforce. In fact, the most common form of communication on the matter was silence. We suggest this silence indicates a missed opportunity in offering additional support for new mothers to modify their pre-partum smartphone habits, in service of the parent–infant relationship.

## 1. Introduction

Given the altricial state of newborn human infants, a secure attachment relationship is advantageous for their optimal development. The formation of a secure attachment is most likely to a baby’s parent(s) [1]. Further, a secure attachment is most likely when the parent is sensitive to a baby’s cues, emotionally available, and attuned to the infant’s needs. In today’s world, ubiquitous parental smartphone use has the real potential to disrupt secure attachment formation by interrupting this sensitivity, availability, and attunement [2,3]. This disruption has serious public health implications, including increased risks of mental health issues [4], behavioural problems [5], and long-term developmental challenges [6] in children.

While parental smartphone use may hold benefits for parents, none are evident for the developmental outcomes of their infants when caregivers use their phones during childcare practices such as infant feeding. The younger the infant, the greater their needs and the greater the risks associated with caregiver distraction by the smartphone, given the unique neurobiological state of a newborn and the lifelong implications of parental co-regulation [7].

For adults, smartphone use can be helpful if it complements the concurrent non-digital activity [8], or when the smartphone user themselves regulates the time spent on their device. This regulatory ability seems to determine whether parents perceive a reduction or an increase in stress as a result of smartphone use [9]. For new mothers, the time spent using social media correlates with self-reported rates of loneliness [10]. A third factor in whether phone use will fulfil or drain seems to be the purposefulness or otherwise of the phone use, with mindless scrolling especially problematic for the well-being of both the phone user and whoever else is present [11].

Infants, whose parents may be using smartphones during 27% of their parent–child time [12], are at risk of experiencing distracted care. Recent meta-analyses found strong evidence that increased parental smartphone use during caregiving is negatively associated with a range of developmental domains, including children’s social–emotional development [13,14]. This is, perhaps, unsurprising given that smartphone use has been found to contribute to a parent’s blank facial affect, mimicking the ‘still face paradigm’ [15] and causing physiological stress for infants [16]. Smartphone use can also contribute to facial expressions, which have meaning to the smartphone user but not those observing, which can be challenging for infants, given their need for predictable parental signals for optimal neurodevelopment [4]. Further, parental smartphone use can interfere with a child’s cognitive development, including their ability to learn language. As one example, the number of audible notifications on a parent’s smartphone negatively correlates with a child’s vocabulary at 18 months [17].

Moderating use of the smartphone is not straightforward. The allure of smartphones is intentional, with the use of so-called sticky design [18], and persuasive tech [19], as well as purposeful strategies to increase a user’s time on the device [20]. For example, Hiniker [21] writing from her experience in technology design, describes “a variety of *dark patterns* in existing commercial technologies, that is, design approaches that serve the product and the company at the expense of the user” (p. 36, emphasis in original). Hiniker cites design strategies that “seek to erode users’ ability to self-regulate their use” (p. 36), despite that same ability determining the stress associated with parents’ use of the smartphone [9].

Recognising the commercial determinants of health [22], it is necessary to approach this subject with compassion for parents, rather than scolding. After all, “It is unreasonable to design services to be compulsive, and then reprimand [people] for being preoccupied with their devices.” [23] (p. 5). Nonetheless, reduced time spent on the smartphone would benefit mothers themselves [10,24] and it is well documented that unfettered smartphone use during caregiving is suboptimal for babies [14,25,26,27].

Although parental absorption in media and mobile devices has been found to significantly predict attachment insecurity [28], it would seem that new parents are largely unaware of this information [29]. In one Australian study of smartphone use in birthing suites, Lewis, et al. [30] found that only 17% of women “felt their phone would impact on quality time spent with their baby” (p. 11). Meanwhile, research that interviewed parents of infants in the United States of America (USA) suggested that parents expressed a general awareness of the potential negative impact on child development as a result of parental phone use, but that they “seemed relatively unconcerned” about this [31] (p. 77). Furthermore, it is unknown whether parents are receiving advice about their own smartphone use from perinatal professionals. Although some parents may mistrust such advice [32], others are likely to appreciate more guidance [33].

Either way, any discussions with perinatal professionals seem likely to focus on information about children’s use of such technologies, for example, if referring to the screen time recommendations as published by the World Health Organization [34] or the American Academy of Pediatrics (AAP) [35]. However, to the best of our knowledge, no country’s well-child checks or perinatal care regimes currently include discussion about the need for purposeful smartphone use by parents, despite urging from researchers [36] and despite parental screen habits being powerfully influential in children’s own screen habits later [37,38,39]. An intervention in Germany with paediatricians to prevent dysregulated screen time by children under three years of age is underway; however, it includes consideration of parental use [40].

Focusing on first-time parents at the transition to parenthood, this study acknowledges the importance of this life stage, especially with regard to contemplating change around health behaviours [41,42]. Such health behaviour change can include ‘parent-for-child’ health behaviours [43], as in modifying parental smartphone habits. In this study, we explore primiparous mothers’ responses to the following data collection questions asked during semi-structured interviews: Who (if anybody) has talked to you about your postpartum smartphone use and the implications for child development? Who (if anybody) would you prefer to have such conversations with?

## 2. Materials and Methods

This article is part of a larger project that co-created an intervention to support the purposeful use of smartphones by primiparous women during the transition to parenthood. That project measured the intervention’s efficacy and sought to learn about smartphone-related conversations initiated by the perinatal workforce [44].

### 2.1. Study Design

The research described here used qualitative semi-structured interviews with 12 first-time mothers. Semi-structured interviews encourage focus on specifics while allowing for exploration of ideas that may come up spontaneously during interviews [45]. These interviews focused on individual experiences [46].

Questions posed to participants included the following (extension questions appear in brackets):

*When I share that the mission of this research project is to support new mums in creating healthy smartphone habits, what is your first instinct about that goal? What thoughts does that bring to mind? (Why do you say that? Can you think of an example for me?)*

*I’m also trying to figure out who would be the best person to have these conversations with new mums. Before our conversation today, I wonder if you had discussed with anyone your own smartphone habits during your pregnancy? What about after the baby arrived? (What did you talk about? What impact did that have on you? Some new parents turn to their doctor … how do you think that would go in your case? Who else might be appropriate?)*

*When you think about all the mamas you know, who do you think they would best hear that sort of thing from? (Why do you think you would listen to that person?)*

These interviews were approached with an awareness that the principal investigator (and interviewer) walked a fine line between being an insider (children’s advocate, mother) and an outsider (older than the interviewees by 20–30 years, not active on social media). While it may be the case that this absence from social media could have coloured questioning or interpretation of data, this dual positionality is not necessarily problematic. As McIntyre [47] highlights in their work, similarities between researcher and participants offer a framework of trust and communication, while differences provide opportunities for mutual development and learning.

### 2.2. Theoretical Framework

Relevant to this paper are multiple theories of human development, and a critical pragmatist stance [48]. In health research, a pragmatist selects research methods in response to issues at hand [49]. Adopting a critical lens has the dual aim of avoiding a “power-blind approach” [50] (p. 977), and allowing for critique of the attention economy, fuelled as it is by business practices that keep users’ focus on their screens at all costs [51], without regard for whether infants are present. This acknowledgement of power dynamics in research reiterated the importance of allowing research participants to choose the location and time for interviews, and the vital need to prioritise supporting the mother-infant connection during interviews (for example, pausing questioning in response to babies’ needs and respecting mothers’ attention).

In terms of human development, Attachment Theory [52], the Polyvagal Theory [53], Bronfenbrenner’s Bioecological Model [54] were influences on this research, and a brief overview of each follows.

Attachment Theory offers an explanation for the essential role of social connection in human development [55], with the need to attach described as a human motivation as fundamental as humans’ drive for food or sex [56]. Given that maternal smartphone use has negative impacts on the formation of a secure attachment, it is a useful foundation for understanding the relevance of paying attention to smartphone habits in babies’ presence.

It could be said that the Polyvagal Theory [53] is a biological explanation for the mechanics of attachment. The Polyvagal Theory highlights that humans’ constant monitoring for cues of risks and safety begins in infancy. This vigilance can best be calmed by loving and soothing connections, enabling the regulation of emotional and bodily states. In the Polyvagal Theory are descriptions of the specific neuroanatomical pathways described as face-to-heart [57], emphasising the role of a caregiver’s facial expression in fostering a sense of safety in an infant. In this way, parental interaction influences a baby’s ability to feel safe and calm, which impacts their access to the health-enhancing state of homeostasis [58]. Conversely, parental smartphone use—which blunts the facial expression and affect of the user—has been linked with vagal withdrawal in young children [59].

Finally, this study leans on Bronfenbrenner’s Bioecological Model of Human Development [54]. In the Bioecological Model, child development is impacted by a nested arrangement of tiers of influences and structures. At the centre of the model sits a person, a biological being with unique traits. Surrounding that person is the innermost tier, called the microsystem (consider here the influence of whānau/family). Next is the mesosystem, which encompasses the influences of professionals and the community. Then, the macrosystem, where policy and structural factors influence development. This includes the internet and all that smartphones offer. The Bioecological Model is a useful contribution to the theoretical framework for this work in that it is a tool to visualise the influence of commercial interests on child development.

In considering these theories of development, they could be thought of as aligning with a relational analytical lens. Therefore, the overarching influence of the theoretical framework is the prioritisation of an infant’s primary relationships as being crucial for their optimal development.

### 2.3. Recruitment and Participants

Participants were recruited in two waves, in alignment with the multiple phases of the doctoral research project from which this paper arises [44]. All interviewees were primiparous mothers, comprising a total of 12 participants. The first set of interviews was undertaken with six primiparous mothers of babies, all within the area of Christchurch, Aotearoa|New Zealand. These women were recruited with the support of Plunket clinics in Christchurch and the wider Canterbury region. Plunket is a charitable organisation that provides support services for children and families, including well-child checks (see: www.plunket.org.nz accessed on 1 May 2022). Convenience sampling of friends-of-friends or colleagues-of-colleagues resulted in further eligible candidates being recruited for interviews.

The second set of interviews involved purposive selection of six women who had been involved in the quantitative intervention portion of the doctoral research project from which this paper arises. These primiparous mothers had previously indicated that they were open to being invited to participate in these interviews.

In terms of sample size, data saturation was not the goal, as it need not always be in health research [60]. Instead, it was determined in response to the specifics of this broader project and its exploratory sequential design [44]. The criteria for participation were that the women were aged 18 or older, first-time mums, smartphone users, and English speakers. In all cases, the women emailed the principal researcher to enquire about the study, and having had their eligibility established, they were emailed a participant information sheet and consent information. A koha, or gift, of a grocery voucher was offered to them upon completion of their interview.

### 2.4. Data Collection

Data were collected through two sets of semi-structured qualitative interviews. The first round of interviews (six) took place in late 2022, and were conducted face-to-face at either the women’s local Plunket clinic or in their own homes, as was convenient for them and their babies. The babies in this group were aged between 2 and 9 months.

The second round of interviews (in mid-2023) all occurred online Via Zoom, as the six participants came from all over Aotearoa|New Zealand. For this phase, the babies were aged between 1.5 and 3 months old.

In all cases, a digital voice recorder was used to capture interview data, and responses were analysed as one data set. The women were invited to choose how they, and their infants, would be referred to in this research. Some decided upon pseudonyms, others selected their given names.

### 2.5. Data Analysis

Reflexive thematic analysis is “an interpretive method firmly situated within a qualitative paradigm and as such a viable analytic option for qualitative health researchers” [61] (p. 2012). This analytic method does not prescribe a specific theoretical framework, in contrast to other approaches to qualitative data analysis—for example, interpretive phenomenological analysis [62]. Instead, reflexive thematic analysis encourages researchers to select theory and epistemology, enabling the use of Bronfenbrenner’s Bioecological Model [63] as the theoretical lens for generating, organising, and describing themes.

Upon completion of each interview, the sound files were uploaded to a password-protected computer and were manually transcribed. A hard-copy printout of each interview allowed a tactile and tangible immersion in each woman’s words [64], which were read and reread “from beginning to end, as one would read a novel” [65] (p. 1280), before coding commenced.

Next, the stories shared by the new mothers were coded deductively, and early in the process, the principal investigator (MM) met with our team’s qualitative research specialist (RD) in order to compare coded transcripts. This enabled the sharing of codes and a discussion of similarities or differences, demonstrating a commitment to dependability and trustworthiness in sharing these data [66]. The data from the two phases of interviews were combined after coding, given the similarities in their content.

### 2.6. Ethical Considerations

This study was registered with the Australian New Zealand Clinical Trials Registry (ANZCTR) in May of 2022 (Registration Number ACTRN12622001184763). The Health and Disability Ethics Committee (HDEC) provided approval in 19 July 2022 (Ref: 2022 EXP 12220). These successes enabled an expedited trip through the University of Canterbury’s Human Research Ethics Committee process, and initial approval was gained in August 2022. Upon acquisition of these approvals, the study gained ethical approval from Whānau Āwhina/the Royal New Zealand Plunket Society (also known as Plunket) to partner with them for participant recruitment.

Within the various ethical considerations of undertaking this project was an awareness that the transition into parenthood is recognised as being a stressful life stage [67,68,69,70]. As such, the information sheets that accompanied the consent forms reminded women that they could remove themselves from the study at any time and included resources for mental health support.

## 3. Results

Overall, regarding conversations about smartphone use within the mesosystem, the experience of most of the women here seems to have been one of silence—although with exceptions. An interviewee named Ainslee was the lone example of having been invited to consider this topic during pregnancy. She said: “I feel like it might have got briefly mentioned at the antenatal Plunket, just about the use of smartphones versus being present. But very, very briefly, I don’t think there was a long discussion about it, at all.”.

Meanwhile, Anna had heard a brief mention in a postpartum Plunket education setting. She shared: “It was all about mutual gaze, and this is how you should be putting down your phone, and they did talk about that…”. Eva had a slightly different take on her experience of a postpartum Plunket class, saying:

*“Um, y’know you go to Plunket class and they’re like “make eye contact with your baby, do this and this” but they don’t ever talk about, like, “so, if you’ve got your phone, use it like this, and not like this”.”*

Otherwise, none of the interviewees remembered having had smartphone or technology-related conversations with professionals, on either side of their transitions to parenthood. Nicole summarised this finding as: “No. I’ve never heard … I’ve never heard anyone discuss it before.”, while Rebecca indicated that: “literally nothing has come up around, like, screentime, or especially breastfeeding and, like, things like that … um… no. It’s interesting! Because it’s such a big part of our lives.”.

Sarah added to this with her observation that:

*“No. No. No one’s raised that with me directly and sort of forthrightly …in fact, interestingly, on the flip side, smartphone use and entry to motherhood has been encouraged. So, for example, when I was doing the breastfeeding course at one of the local hospitals, they encouraged me to download an app. Yeah, so I think actually smartphone use was encouraged.”*

Sarah’s perspective of having been encouraged to go online by professional entities was echoed by Anna, who brought this idea up at two points during our conversation:

*“… you should be looking on your phone … you know I kind of felt that. Like ‘why aren’t you on that (Plunket) Facebook group?… Oh, no you just go on Facebook’. And: ‘have you got the app?’ … y’know, those kind of things? Like, ‘well, look at it online and then you can join.’”*

At a separate moment in the conversation, Anna said:

*“A lot of providers, I think, are giving a lot of emphasis to online apps and websites to get their information. And I know with the Plunket nurse when she came to see me, she was like “for your next visit, you just need to go online” or “you need to go on to this app and um … to get into these groups”.”*

There was a range of professional services encountered by the women (Plunket, midwives, antenatal groups—both private and public, including the Māori provider Te Puawaitanga, nurses providing six-week immunisations, providers of Ministry of Health-funded Well-Child checks, breastfeeding support at the hospital). However, there was no evidence of a coordinated effort to provide messaging about the need for mindful smartphone use in a baby’s presence from any of these groups. Conversations with women during the perinatal period about their smartphone use seem to have been Ad Hoc, with Ainslee one of two women who thought it “might have got briefly mentioned”.

Such silences may be partially demonstrated by the following comment from Jess, who said about smartphone use: “… well, I’m actually a healthcare professional as well, but it’s not something I would ever bring up to a mother …”.

Jess’s unwillingness to initiate such a discussion contrasts with a desire expressed by multiple interviewees for education and conversation on both sides of a baby’s arrival. The value of this pre- and post-partum approach is reiterated by research [71]. This notion was described by Sarah as such:

*“… for me it would have been helpful to have some pre-education. And then some follow up kind of education on it … an antenatal class would have been a helpful place to be exposed to this kind of information, initially, and then I know that Plunket have been really helpful, so that would be another avenue postnatally, … that I think … either just to check in on how it’s going, how that use is going … once that seed, and the idea had been planted … how it’s going. And how it’s making the person feel. … Midwives are also another avenue that, I guess, are in contact with mothers prior and post birth, who can have those kinds of conversations as well.”*

While Anna shared an aspirational vision for professional engagement extending beyond education about technoference or other smartphone-related phenomena:

*“Not just … smartphone or media use but so much more … you know, it’s another layer to add … a really important topic that is obviously not talked about enough or known enough about … it would be the midwives driving that, I suspect, if it was in pregnancy … And I think, for New Zealand … it should be all one hub, …ideally, one lead professional that you see all the way through that you have a trusting relationship … if women had one trusting person that they go to, whether it be the midwife, or Well-Child … Rather than having so many bits and pieces … another layer. Which I think, if this is incorporated into a bigger child health or child wellbeing plan, or model for mums, for new parents.”*

Anna’s vision was that this consciousness-raising work about smartphone use in early parenthood ought to sit within known relationships, for example, with a midwife. Such a perspective aligns with findings that new material should slot into existing contexts rather than create a new entity for engagement [72].

This idea of using existing frameworks to share information about purposeful smartphone engagement would likely work, at least from the parents’ perspective. When interviewees were asked who they would prefer, or think their peers would prefer, to have such conversations with, they had a range of suggested entry points for engagement, all relying on existing contexts. One of the participants, Ainslee, suggested midwives, as they are a person already known to a mother:

*“… midwife could’ve been quite good. Because she could have talked about it pre- and then, coming into the home and saying, y’know, you do have the conversation with your midwife about your mental health postpartum, and that could be part of that sort of follow-through.”*

Sarah also identified midwives as a valuable source of postpartum support, and she had numerous other suggestions:

*“It’s interesting that … you know … for some people, using technology can be the greatest support, like … having a conversation around is it positive for you? Is it negative? What can we do to support you in managing that use, either way? Um … so I think, postnatally, Plunket would be a good option, um … but … I think there are other avenues. Like the six-week vaccinations with the nurses and the doctor when you go in for that check … the midwives.”*

Like Sarah, there were other mums who identified the antenatal period as being an optimal time for hearing information about smartphone use in babies’ presence:

*“Yeah, speaking about myself and my friends, we tried to learn as much as we could while we were pregnant, and a lot of us listen to a lot of audio books, y’know talking about pregnancy and the different stages and all that sort of thing, but … yeah probably around then I would say, because you’ve got more time on your hands.”*

The previous point made by Nicole was reiterated by Laura, who said:

*“…antenatal … that’s when a lot of people pick up their information … because they know nothing. Um … Plunket … I think it depends who your nurse is, … yeah… it depends on your nurse. But I think probably people absorb the most at antenatal, because they don’t have a kid yet and … y’know they want to know everything. Once you have your kid you’re like “oh, shit. I haven’t got time for any of that”. So I think maybe … maybe prior.”*

To summarise, suggestions included antenatal education, including Te Puawaitanga, perinatal care such as midwives, and those who are involved with the family postnatally, such as Plunket or the general practice nurses and doctors who administer immunisations to babies. There are multiple possibilities, but so far, there appear to be few conversations.

This is disappointing, given that women seem ready and willing to have dialogue with the perinatal professionals in their lives. Eva distilled that notion as follows:

*“I think (we) would be open to it. I think … we’re …like “Oh, I’ll take that on board, we’ll see what we can do!”. Um … But, just like bringing it up in conversation … no one brings it up in conversation, so … um, just having it there would be helpful.”*

The feedback from the women interviewed here suggests that there is a long way to go in leveraging the power of the women’s mesosystems to provide information and support in this issue—perhaps first requiring edification for the workforce itself.

## 4. Discussion

### 4.1. Missed Opportunity for Mothers and Babies

The perinatal professionals working with these pregnant and puerperal women do not appear to be initiating conversations or sharing information about the potential for harm to multiple domains of child development resulting from distracted caregiving. This trend of silence echoes international findings [6,25,73,74]. With one exception, all the interviewees’ experiences can be summarised by the participant quote, which informed the title for this paper: I haven’t had anyone talk about tech use, at all. In contrast, most mothers expressed a desire for more access to information about how to manage the interaction of their matrescence and their digital lives.

Such information could go further than discussion of the impacts on infant development; it could also offer evidence-based support for parents about how to support rather than harm their own mental health. Such advice could include a caution about ‘mindless scrolling’ [11], the benefits of limiting time on social media [10], and supports for enhancing digital literacy to mitigate the negative effects of social comparison online [75].

### 4.2. Invisible Infants?

This may be a case of research moving faster than practice, with a need to close the gap between evidence and routine clinical practice [76]. This would certainly seem to be the case in the examples cited by Sarah and Anna, where they felt that they were being encouraged to go online, without any caveats for considering their own mental health or their infants’ relational needs in choosing how or when to do so.

Perhaps it is little wonder clinicians send women online without such a caveat: they may be heeding the incomplete advice of academics and journal authors in the research literature. Examples abound of academics recommending that professionals create online content for new mothers, without any accompanying caution about the wisdom of encouraging purposeful engagement with the smartphone (for example, waiting until children are asleep before using the smartphone, if possible), or limiting use—especially in the presence of their children [77,78,79,80,81].

This tendency may be part of a larger issue at play in the world of academic research, where the “subjective experience of infants is seldom considered in research directly concerning them” [82] (p. 70). A lack of consideration for infant experience and developmental trajectories in the research community could trickle down into, or perhaps echo, an unintentional thoughtlessness on the part of the clinical workforce. This is despite contemporary encouragement to assume an early-onset emergence of consciousness in infants [83].

### 4.3. The Case for a Multilayered Response

For new mothers to change their pre-partum smartphone use habits at the transition to parenthood in deference to the developmental needs of their infants, they would need support at all layers of their lives—all the layers of the Bioecological Model. This would include the support of consistent and ongoing messaging from the perinatal professionals who serve them, which may require a concerted effort to offer training or education to that clinical workforce. This is challenging yet vital work, given that the research in this field is fast-moving and rapidly accumulating. Training for this workforce could include resources to integrate smartphone-use discussions into antenatal education and the development of brief conversation guides for providers of well-child services.

Such training and resource development would require funding, and it is relevant to ask whether policymakers have the knowledge or the will to fund such initiatives. Admittedly, it may be challenging to research this possible knowledge gap in legislators, at the next layer of the Bioecological Model. Given the budget cuts to the public service that are ongoing in Aotearoa|New Zealand at the time of writing [84], such investment seems unlikely, at least here and now.

If policymakers’ knowledge base about—and desire to tackle—this issue was found to be sufficient that such funding of healthcare workforce training could be undertaken, this may mean policymakers were similarly motivated to regulate the practices of online actors in some way. They could insist on an ethical code of conduct for those who put a technological layer between mothers and infants, including a requirement for medical or developmental oversight in any apps that target those who are pregnant or parenting. At present, there seem to be fewer than 3% of apps with any such professional oversight [85].

### 4.4. Infant Feeding

A need for such training for the perinatal workforce may explain the surprise felt by one interviewee that nobody had discussed smartphone use and breastfeeding with her. This was despite phone use during feeding being something she identified as a big part of the life of a new mother. Certainly, infant feeding routines are of particular interest here, given that they hold “both nutritional and social significance” [86] (p. 3). Research indicates that smartphone use during infant feeding routines is now commonplace [29,87,88,89]; this is uncharted territory for the parent–infant relationship.

Mothers distracted by their phones during breast- and bottle-feeding routines are more likely to miss infant cues of satiation, leading to overfeeding, which can contribute to issues with obesity later in life [88]. Further, there are evolutionarily adaptive habits that tend to accompany infant feeding [90] such as affectionate touch, shared gaze, conversation, and song. These are at risk of being interrupted by parental distraction with smartphones [87,89] as they busy mothers’ hands and minds.

Establishing healthy smartphone habits during early feeding routines could ensure that the 8–12 feeds per day [91] typical of early caregiving are experienced—by mother and baby alike—as opportunities for intimate relational connection. The potential for this as being a missed opportunity stands out when positioned alongside the revelation from an interviewee who was also a healthcare professional: that she would never bring up the topic of screen use during caregiving to a mother in her care. This declaration, and the intentionality of her silence, seem incongruous with the effects of smartphone use on the developmental trajectories of infants and the mental well-being of their mothers.

### 4.5. Modifiable Factor: Distracted Attachment

Infant development is being compromised by the current, normalised patterns of smartphone use while caregiving, and there do not appear to be coordinated responses from any healthcare agencies to respond to this threat (at least, in Aotearoa|New Zealand). Young families at the transition to parenthood are not currently being offered information or support about this topic. This is curious, given that parental smartphone use is associated with a range of suboptimal developmental outcomes for children, and that it is a modifiable factor [14].

Secure attachment relationships for citizens are worth paying attention to, at the macro level, down to the central circle of the Bioecological Model. A secure attachment relationship is irrefutably valuable. It contributes to positive trajectories across multiple developmental domains, as well as providing protection from toxic stress and resilience in the face of trauma throughout the lifespan [92,93].

Formation of a secure attachment relationship depends upon maternal sensitivity as a key precursor, and maternal sensitivity has been demonstrated to suffer when mothers are distracted by their smartphones [94,95,96]. Increased parental smartphone use is directly associated with reduced patterns of secure attachment [25]. Further, the rapid neurobiological growth of children during their early lives holds lifelong implications [97].

As such, responsive caregiving could be considered a public health priority [98] and physicians could consider treating a parent–infant relationship as a patient, rather than either individual [99]. Therefore, favourable experiences and supportive relationships for infants ought to be of interest to clinicians and policy makers alike, given the fiscal and human value of early, preventative interventions [100]. Addressing the “social pollution” [101] (p. 35) of parental smartphone use during care would go some way to support these goals.

The silence from the perinatal workforce represents a missed public health opportunity to support early parent–infant relationships. Without proactive conversations, new mothers may continue habits that undermine emotional attunement and secure attachment [95], potentially contributing to long-term developmental and mental health risks for their children [102].

### 4.6. Strengths and Limitations

Strengths of these semi-structured interviews include their demonstrated commitment to respecting mothers, infants, and their dyadic connection. It is a strength of this study that consideration of, and respect for, infants was a connective thread in the motivation, design, implementation, and documentation of the project. It should not be a radical notion to consider an infant’s eye view when reading research or designing a study that involves infants, and yet such consideration rarely seems to be the case.

In terms of limitations, the review of literature was limited to work published in English, despite these phenomena being global in nature. Also, there was the risk of social desirability bias at play in data collection [103], especially given New Zealanders’ documented tendency for indirectness in communication [104].

It could be considered a limitation that this work has combined data from two different sets of interviews, undertaken with a gap of several months in between, and a subtly different population of women. While the combining of these data was appropriate given the commonalities in the women’s stories, the existence of two waves of data collection was in response to the multiphase, exploratory sequential nature of the doctoral project, rather than an aspirational feature of the study’s design.

Further, the modest sample size could be considered a limitation, as could the study’s focus on mothers only. This maternal focus was a reflection of the pragmatic constraints of doctoral study, despite an understanding that best practice would have involved other family members. Similarly, the usefulness of this study would have been enhanced if it had included perspectives from interviews with members of the perinatal workforce. These considerations, particularly for wider family and perinatal professionals, would be appropriate inclusions for future research.

## 5. Conclusions

Semi-structured interviews with primiparous mothers revealed a silence from the perinatal workforce regarding information about the potential for harm brought about by technoference during caregiving. Reduced or purposeful use of the smartphone is associated with benefits for both maternal mental health and infants’ developmental trajectories. However, there is no apparent coordinated effort on the part of the numerous healthcare professionals in new mothers’ lives to communicate this, despite mothers’ expressed desire for such information.

In health research and practice, when there are so many competing needs, it can be hard to know where to look and what to prioritise. Families need appropriate housing, food security, and clean air. They need fresh water, reliable healthcare, and opportunities for play. However, we must not let these irrefutably important factors obscure the threat that rampant technoference poses to optimal infant (human) development. The rapid embrace of digital devices into every crevice of our daily lives is at odds with babies’ fundamental needs, and we are only just beginning to see what that will mean for whole cohorts of people. We argue that it is incumbent upon ante- and post-natal healthcare providers to initiate this conversation with the parents with whom they engage.

## Data Availability

The data presented in this study are available on request from the corresponding author (miriam.mccaleb@canterbury.ac.nz). The data are not publicly available due to ethical concerns regarding the publication of interviewee transcripts in a public repository. Specifically, when the data were collected, participants only gave consent for the findings to be published anonymously for educational and research purposes. Making the data public would violate this consent.

## Abbreviations

The following abbreviations are used in this manuscript:

AAP	American Academy of Pediatrics
ANZCTR	Australia/New Zealand Clinical Trials Registry
HDEC	Health and Disability Ethics Committee
USA	United States of America
